# Molecular Mechanisms of Stem/Progenitor Cell Maintenance in the Adrenal Cortex

**DOI:** 10.3389/fendo.2017.00052

**Published:** 2017-03-23

**Authors:** Antonio Marcondes Lerario, Isabella Finco, Christopher LaPensee, Gary Douglas Hammer

**Affiliations:** ^1^Department of Internal Medicine, Division of Metabolism, Endocrinology, and Diabetes, University of Michigan, Ann Arbor, MI, USA; ^2^Endocrine Oncology Program, Comprehensive Cancer Center, University of Michigan, Ann Arbor, MI, USA; ^3^Center for Organogenesis, University of Michigan, Ann Arbor, MI, USA

**Keywords:** adrenal cortex, paracrine signaling, stem cells, organogenesis, extracellular matrix, tissue homeostasis

## Abstract

The adrenal cortex is characterized by three histologically and functionally distinct zones: the outermost zona glomerulosa (zG), the intermediate zona fasciculata, and the innermost zona reticularis. Important aspects of the physiology and maintenance of the adrenocortical stem/progenitor cells have emerged in the last few years. Studies have shown that the adrenocortical cells descend from a pool of progenitors that are localized in the subcapsular region of the zG. These cells continually undergo a process of centripetal displacement and differentiation, which is orchestrated by several paracrine and endocrine cues, including the pituitary-derived adrenocorticotrophic hormone, and angiotensin II. However, while several roles of the endocrine axes on adrenocortical function are well established, the mechanisms coordinating the maintenance of an undifferentiated progenitor cell pool with self-renewal capacity are poorly understood. Local factors, such as the composition of the extracellular matrix (ECM) with embedded signaling molecules, and the activity of major paracrine effectors, including ligands of the sonic hedgehog and Wnt signaling pathways, are thought to play a major role. Particularly, the composition of the ECM, which exhibits substantial differences within each of the three histologically distinct concentric zones, has been shown to influence the differentiation status of adrenocortical cells. New data from other organ systems and different experimental paradigms strongly support the conclusion that the interactions of ECM components with cell-surface receptors and secreted factors are key determinants of cell fate. In this review, we summarize established and emerging data on the paracrine and autocrine regulatory loops that regulate the biology of the progenitor cell niche and propose a role for bioengineered ECM models in further elucidating this biology in the adrenal.

## Introduction

In vertebrates, adrenal steroid hormones are effectors of different adaptive responses to oscillations in the organism’s internal and external environment, broadly referred as *stress*. Adrenal steroid hormones serve to modulate a wide range of processes that are central to physiologic response to stress, including energy metabolism, immune response, electrolyte homeostasis, and fluid balance. The three main classes of adrenal steroid hormones, mineralocorticoids, glucocorticoids, and androgens, are produced by the adrenal cortex under the tight regulation of distinct and independent endocrine regulatory loops: the renin–angiotensin–aldosterone system (RAAS) and the hypothalamus–pituitary–adrenal (HPA) axis. The ability of the adrenal gland to respond independently to these endocrine signals is dependent upon subpopulations of steroidogenic cells with distinct morphological and functional characteristics that are localized in specific concentric compartments (zones) of the cortex. In humans, three histologically distinct zones are evident: the outermost *zona glomerulosa* (zG), the intermediate *zona fasciculata* (zF), and the innermost *zona reticularis* (zR), which are responsible for the production of mineralocorticoids, glucocorticoids, and androgens, respectively (1). Although the morphological and physiological aspects of the adrenal cortex have been relatively well characterized, the regulatory mechanisms responsible for the establishment and maintenance of the three zones are not fully understood.

In the last few years, sophisticated molecular techniques, such as lineage tracing, and genetically modified animals have significantly contributed to our understanding of the embryonic development and homeostasis of the adrenal cortex, illuminating key molecules and signaling pathways that are implicated in these processes (discussed below in Sections “Progenitor Populations in the Adrenal Gland” and “Signaling Pathways and Adrenal Progenitors”). Accordingly, the Wnt and the hedgehog pathways have emerged as major paracrine factors that regulate both organogenesis and homeostasis of the gland. Both are essential for the establishment and maintenance of an undifferentiated population of steroidogenic precursor cells in the periphery of the organ that continuously replenish the cortical cells of the three zones throughout life (discussed below in Sections “Progenitor Populations in the Adrenal Gland” and “Signaling Pathways and Adrenal Progenitors”). While the effectors of the RAAS and the HPA axis [angiotensin 2 and adrenocorticotrophic hormone (ACTH), respectively] are considered primary endocrine mediators that promote activity of adrenocortical steroidogenic cells (2–4), recent data support that the *microenvironment*, comprised of the extracellular matrix (ECM), its associated proteins, and several paracrine and autocrine factors, are also major determinants of cell behavior and fate (discussed below in Sections “Signaling Pathways and Adrenal Progenitors” and “The Adrenocortical Progenitor Niche”). Emerging data support an important role for non-steroidogenic/mesenchymal-like cells that lie in the adrenal capsule in the maintenance of the adrenocortical progenitor pool (5–8). This review focuses on the role of microenvironmental factors and signaling pathways that contribute to the maintenance of the adrenal cortex. We will discuss emerging data on the role of specific paracrine and autocrine loops between capsular/stromal cells and cortical cells in establishing a specialized microenvironment that supports and maintains the adrenal progenitors throughout the life—the *niche*—and how this signaling contributes to the anatomical and functional zonation of the adrenal cortex.

## Developmental Origins of the Adrenal Cortex

The adrenal cortex originates from cells of the celomic epithelium, which in the mammalian embryo is composed of a single squamous cell layer that covers the external surface of the viscera and the inner surface of the body wall. Around embryonic day 9.5 (E9.5) in mice, and during the fourth to sixth weeks of human gestation, a thickening of the celomic epithelium between the urogenital ridge and the dorsal mesentery forms the adrenogonadal *primordium* (AGP). At the eighth week of human gestation (E10.5 in mice), the AGP divides into dorsomedial and ventrolateral portions, giving rise to the adrenal and gonadal *primordia*, respectively (1, 9–11).

Between the weeks 8 and 9 of human gestation (E13 in mice), the *adrenal primordium* is invaded by cells of the neural crest that coalesce centrally to form the adrenal medulla (12). Subsequently, the *adrenal primordium* is surrounded by mesenchymal cells, which will ultimately form the adrenal capsule (13). At this point, compartmentalization of the adrenal cortex into two structurally distinct areas is evident: a central area, comprised of large polyhedric eosinophilic cells referred as the “fetal zone,” and a peripheral zone adjacent to the newly formed capsule comprised of small and basophilic cells, referred to as “the definitive zone.” While in mice this compartmentalization is subtle, in humans, the fetal zone predominates over the definitive zone, constituting up to 80% of the adrenal mass by the end of the gestation (9, 14). In addition, ultrastructural studies in humans have demonstrated the presence of a third zone, referred as the “transitional” or “intermediate zone,” which has intermediate morphologic characteristics between the fetal and the definitive zones (9). It has been suggested that after mid-gestation the transitional zone has the capacity to synthesize cortisol (9, 15). By the week 30 of human gestation, the definitive and the transitional zones have morphological features that resemble the adult zG and zF, respectively (16). In humans, the fetal cortex starts to regress by apoptosis soon after birth, completely disappearing after a few weeks (9). In mice, definitive evidence about the presence of a transient fetal zone was provided by the identification of the fetal adrenal-specific enhancer (FAdE), which is only active during early fetal development (see below). The differentiation process of the human adrenal cortex continues until the onset of puberty, when the definitive cortex completes its organization into the three distinct histologic zones that characterize the adult cortex of human and higher primates (14). While the zG and the zF are evident at birth, the androgen-producing zR only starts to form a few years later, marking the onset of the adrenarche, which is the earliest stage of sexual maturation and a precursor of puberty (17).

A transcription factor critical for adrenocortical development and homeostasis is steroidogenic factor 1 (SF1, also known as adrenal four-binding protein or nuclear hormone receptor Ad4BP, encoded by the gene *NR5A1*). All cells that belong to steroidogenic lineages of the adrenal and gonads express SF1, including subpopulations of long-term retained progenitor cells in each organ (8, 18). Therefore, SF1 expression defines the identity of these cells and commitment to steroidogenic differentiation (14, 19, 20). The expression of SF1 is detectable early in fetal life, between the AGP formation and the ultimate establishment of the adrenal *primordium* (14, 18). Genetic loss of *Nr5a1* or its upstream transcriptional regulators *Pbx1, Wt1*, and *Cited2*, interferes with AGP formation leading to various degrees of adrenal hypoplasia in mice (12, 14, 21–23). While *Nr5a1* is continuously expressed from the time of *adrenal primordium* formation throughout the adult life, during embryonic stages and early fetal life in mice, the *Nr5a1* expression is driven by the fetal adrenal-specific enhancer (FAdE), which becomes inactive when the definitive cortex forms, suggesting that distinct mechanisms sustain *Nr5a1* expression in the fetal and in the definitive cortex (14).

While the enhancer that is responsible for initiation and maintenance of *Nr5a1* expression in the definitive zone is currently not known, lineage-tracing experiments performed by Zubair et al. have shown that *FAdE*-driven-*Ad4bp*-expressing cells are indeed precursors of most, if not all, Sf1-expressing cells of the definitive cortex (14). It has since been demonstrated that after extinguishing FAdE-dependent SF1 expression, some cells become embedded within the coalescing capsule and later reemerge as FAdE-independent SF1-expressing cells during the formation of the definitive cortex (24).

Steroidogenic factor 1 not only defines the identity/specification of the cells of the definitive cortex but also serves as a key regulator of hormone-dependent steroidogenesis, the hallmark of a differentiated adrenocortical cell. In this regard, the effects of ACTH on differentiation and steroidogenesis are partially mediated by an SF1-induced transcription of steroidogenic enzymes that defines a cortisol-producing cell (25). It remains unclear, however, how a subpopulation of SF1-expressing cells are spared from the pro-differentiation effects of ACTH, preserving their undifferentiated state. One clue to such regulation comes from studies of DAX1, an atypical nuclear receptor that is encoded by the gene *NR0B1* and expressed preferentially in the peripheral/subcapsular cortex. DAX1 functions as a repressor of SF1-mediated transcription and has been shown to have an important role in the maintenance of the undifferentiated state (lack of steroidogenesis) of the adrenocortical progenitor population (26). Studies examining the consequences of genetic loss of DAX1 in the adrenal revealed an unexpectedly hyper-functional zF in younger mice, characterized by an increase in cell proliferation and steroid production. Surprisingly, as the animals aged, they developed adrenal hypofunction consistent with an abnormal premature differentiation of the progenitor cell pool that ultimately resulted in its exhaustion (27, 28). In the next section, we will briefly summarize studies that have begun to explore the molecular and cellular processes that underlie the homeostasis of the progenitor cell population.

## Progenitor Populations in the Adrenal Gland

One of the first reports regarding adrenal regeneration in rats describes the restoration of the adrenal cortex 6 weeks after unilateral adrenal enucleation, a process that involves the removal of the inner content of the adrenal gland (including the medulla), while leaving the capsule and underlying subcapsular cells intact. This observation suggested the presence of an adrenocortical stem and/or progenitor cell population located in the periphery of the adrenal gland (29). The notion of a renewing progenitor population in the adrenal cortex was corroborated by the observation that transplants of bovine adrenocortical cells underneath the kidney capsules of adrenalectomized immunocompromised mice can give rise to adrenocortical tissue with steroidogenic properties (30, 31).

The presence of a progenitor cell population in the periphery in the adrenal gland that gives rise to all the differentiated cell types within the different cortical zones is consistent with a homeostatic model of centripetal migration and differentiation. This idea was first hypothesized in the 19th century, based on histological observations of gradual changes in morphology observed in cells between the capsule and the medulla (32, 33) [reviewed in Ref (34)]. Over the years, different studies have shown that, under physiological conditions, proliferating cells are located preferentially in the outermost layers of the cortex, in the subcapsular region (35, 36). Also, it has been demonstrated that peripheral cortical cells are centripetally displaced until they reach the cortical–medullary boundary and become apoptotic (35, 37). Moreover, studies performed in chimeric and transgenic mosaic rats and mice have shown a radial variegated pattern of the reporters extending from the outer cortex to the cortico-medullary boundary. This observation is consistent with a peripheral clonal origin (38–40). Definitive genetic evidence for a centripetal conversion of adrenocortical cells between concentric zones was finally provided by cell-lineage tracing (8, 41). In one of these studies, transgenic mice were created with a Cre recombinase gene inserted at the *Cyp11b2* locus, in which *Cre* was expressed only in terminally differentiated zG cells (41). When these mice were crossed with mice expressing the *R26R^mT/mG^* reporter, GFP-positive cells that occupied the zG upon *Cyp11b2-Cre* expression, eventually populated the entire cortex. Over time, an increasing number of centripetally located *Cyp11b1*-expressing cells were found to express GFP, indicating that peripheral zG cells underwent lineage conversion to more centripetal zF cells. In 12-week old mice, nearly the entire cortex was comprised of GFP-positive cells. Although these existing data strongly support the existence of a progenitor population localized in the periphery of the gland that gives rise to all the steroidogenic cells types of the cortex through a process of centripetal displacement/migration and differentiation, the molecular mechanisms that govern this process are not completely understood.

Further studies have provided clues on the molecular fingerprint of the progenitor cell populations and the signaling pathways that regulate their renewal and differentiation. Specific paracrine/autocrine signaling pathways are activated in zonally restricted patterns that reflect the different cell subpopulations that are hypothesized to play an active role in organ homeostasis. The most important and well characterized are the Wnt and the hedgehog pathways. Additionally, a cross-talk between cortical cells and capsular stromal cells regulates the activity of these pathways. Below, we briefly summarize these findings.

## Signaling Pathways and Adrenal Progenitors

### Wnt Signaling Pathway

The mammalian wingless-type MMTV integration site (Wnt) signaling pathway is one of the most studied pathways in developmental biology, playing important roles in organogenesis, homeostasis, and stem cell biology (42). Wnt ligands represent a large family of highly conserved morphogens that are characterized by repetitive cysteine residues. Secreted Wnt ligands bind to receptors of the Frizzled and lipoprotein receptor-related protein families on the cell surface. Through several cytoplasmic components, the signal is either transduced through β-catenin, which enters the cell nucleus and complexes with T-cell factor/lymphocyte enhancer factor (TCF/LEF) family of transcription factors to activate transcription of Wnt target genes (canonical pathway), and/or activates the non-canonical Wnt pathway to regulate planar cell polarity and calcium signaling (42). In the mouse adrenal gland, β-catenin expression is first observed at E12.5 in Sf1+ cells of the definitive cortex in a scattered fashion beneath the newly formed capsule (43). After E18.5 and throughout life, β-catenin protein expression becomes more enriched in cells of the zG, which have also been demonstrated to bear active canonical Wnt signaling (43, 44).

Canonical Wnt signaling plays several roles in the adrenal cortex. Recent evidence demonstrates that the population of Wnt-responsive cells is heterogeneous and contains small clusters of Shh-producing cells (considered to be adrenal progenitors, as it will be discussed later) and separate differentiated Cyp11b2-expressing cell clusters (44). Interestingly, despite the well-known role of Wnt signaling pathway in promoting cell proliferation in a variety of organ systems, the Wnt-responsive cells do not appear to be heavily proliferating, implying that any role of Wnt signaling in adrenal homeostatic growth might involve a potential cell non-autonomous mechanism (44).

The Wnt pathway is essential for the establishment and maintenance of the subcapsular progenitor cell population. However, it does not appear to play a role in the early formation of the AGP since expression of Ctnnb1 is first detectable in mesothelial cells overlying the indifferent gonad at around E11.5 and in the adrenal primordium at around E12.5, following their separation from the AGP (43, 45). Studies performed in a *Nr5a1/Cre*-mediated *Ctnnb1* conditional knockout mouse model (*Ctnnb1tm2kem* mice crossed into *Sf1/Cre-high* mice), in which canonical Wnt signaling to adrenal primordium SF1-expressing cells from around E12.5 is reduced or absent reveal a marked decrease in cortical cell proliferation which ultimately results in the complete regression of the adrenal gland by E18.5, despite normal formation of the adrenal primordium at earlier time points (43, 46). Loss of β-catenin in approximately 50% of Sf1-expressing cells and hence a resultant ~50% decrease in β-catenin-mediated Wnt signaling (induced by a low efficiency Sf1/Cre-low driver that allows for a subset of cells to escape recombination) results in a histologically normal adrenal through 15 weeks of age, after which the adrenal cortex undergoes progressive thinning over time and ultimate adrenal failure (43, 46). Consistent with a primordial role of the Wnt signaling in maintaining the progenitor cell population, it has been demonstrated that *Nr0b1 (Dax1)* is a transcriptional target of β-catenin, suggesting that both proteins synergistically act to maintain the undifferentiated state that characterizes the progenitor cell population (28). Together, these evidences suggest that canonical Wnt signaling has a role in establishing and maintaining a pool of adrenocortical progenitors throughout life.

### Hedgehog Signaling Pathway

The hedgehog signaling pathway (Hh) is a conserved evolutionary pathway that is essential for embryonic development and adult tissue maintenance, renewal, and regeneration. Three secreted hedgehog proteins have been described: sonic hedgehog (SHH), desert hedgehog, and Indian hedgehog. These proteins function in a concentration- and time-dependent fashion, controlling several processes ranging from survival and proliferation to cell fate specification and differentiation (47).

Shh is the only Hh family member that is present in the murine adrenal gland, being detected as early as E12.5 and later expressed in a restricted manner in a subpopulation of cells in the outer zG (8). These cells are Sf1-positive and do not express Cyp11b2 or Cyp11b1, the enzymes necessary for terminal reactions that lead to aldosterone and corticosterone production, respectively. While in the mouse adrenal the Shh+ cells are localized in clusters under the capsule, in the rat these cells form a continuous layer localized between the zG and the zF, known as the *undifferentiated zone* (zU) (48).

Lineage-tracing studies that mark all Shh-expressing cells and their descendants reveal that, by postnatal day 12 (P12), virtually all cells of the cortex are derived from *Shh*-expressing cells, consistent with a role for Shh in the peripheral stem/progenitor cells (8). Studies utilizing an inducible *Shh*-Cre recombinase have demonstrated that shortly after recombination in adult mice, Shh-expressing cells, and their immediate descendants are restricted to the periphery of the cortex. Over time, these cells and their descendants form centripetally expanding radial stripes, supporting the hypothesis that the Shh-expressing cells are a progenitor population that gives rise to all other cortical cell populations (8). BrdU-labeling experiments carried out on rat adrenals identified two sites of proliferation in the outer zF and inner zU and between the zG and zU, suggesting that cell proliferation at the periphery of the zU provides cells to both zG and zF (36). However, the proliferative capacity of these cells is more consistent with their identification as progenitors rather than as stem cells as suggested by the authors. Another study on mouse adrenocortical cell proliferation used BrdU pulse-chase labeling to identify a small population of quiescent non-steroidogenic cells located in the outer cortex that may represent a quiescent progenitor population, which is prompted to divide following ACTH stimulation (35). Additionally, this study suggested that two distinct subpopulations of rapidly cycling cells emerged from the progenitor population—the first constituted by cells that proliferate in response to ACTH and migrate inwards and the second constituted by cells that migrate outwards and are less responsive to ACTH. The authors have suggested that these distinct subgroups of cells are committed transient-amplifying cells that are responsible for the maintenance of the zF and zG, respectively (35). Interestingly, another study has shown that Cyp11b2-expressing cells in the zG derived from the Shh-expressing cells undergo lineage interconversion, differentiating into Cyp11b1-expresing cells in the zF as they migrate inwards, suggesting that cells from both zones are in fact derived from the same lineage (41).

In addition to serving as a progenitor cell population in the cortex, Shh-positive cells presumably signal to Gli1-expressing cells that lie within the capsule (48, 49). Interestingly, the capsular Gli1-expressing cells are a mesenchymal-like population that do not express Sf1 (Gli1+/Sf1−) but descends from the fetal FAdE-utilizing Sf1+ cells of the adrenal *primordium* (24). The fate of the descendants of these *Gli1*-expressing cells has been investigated in a transgenic mouse model with an inducible *Gli1-CreERT2* allele. It has been demonstrated that during adrenal development, *Gli1*+ capsular cells behave as stem or precursor cells since they give rise to Sf1+ cells of the early definitive cortex that later become the Sf1+/Shh+ progenitor cell pool (8).

### Other Signaling Pathways

In addition to the Gli+/Sf1− cell population, other mesenchymal-like cell lineages have also been identified in the capsule and the stroma. These cells may also have important roles in the adrenal gland maintenance. Bandiera and colleagues observed a long-retained pool of cells expressing the Wilms tumor protein homolog (WT1) in the adrenal capsule that originates from capsular mesenchymal cells and is characterized by AGP features (50). WT1 is a transcriptional regulator that has important roles in organogenesis of different organs, including the adrenals and gonads. Using a Wt1-Cre recombinase line crossed with a reporter line, Bandiera et al. demonstrated that WT1-expressing cells could give rise to steroidogenic cortical cells in the adult adrenal and the Gli1+ population as well, lending support for a role for WT1 in the activation of *Gli1* transcription.

Wood et al. identified a population of cells in the mouse adrenal that starts to express the transcription factor 21 (*Tcf21*, also known as *Pod1*/capsuling/epicardin) at E9.5 onward. After E14.5, Tcf21 expression becomes predominately restricted to the capsule and further diminishes over time, leaving only a small population of capsular Tcf21+ cells through adulthood. Lineage-tracing experiments revealed that before adrenal encapsulation, *Tcf21*-expressing cells and/or their descendants give rise to both non-steroidogenic capsular cells and steroidogenic cortical cells. On the other hand, the population of Tcf21-expressing capsular cells only gives rise to a population of Sf1− stromal cells that express collagen, desmin, and Pdgfrα and persist throughout adult life within the cortex (24).

Taken together, these studies provide evidence that the capsule is a complex niche comprised of multiple long-term retained cell lineages. The roles of each of these populations in the adult adrenal remain unknown, but recent evidence suggests that signaling coming from the capsule is required for the long-term maintenance of the adrenocortical progenitor cells.

## The Adrenocortical Progenitor Niche

The proper balance of progenitor proliferation and differentiation is crucial, as dysregulation of the mechanisms that regulate the differentiation, proliferation, and self-maintenance of progenitor cells throughout life can result in organ failure. Stem and progenitor cells from different organs and tissues are embedded within a specialized microenvironment, termed the *niche*. Niches are protective locations that support stem cell residence and maintenance. Several components of the niche have been identified, including bioactive compounds, such as Wnt ligands, growth factors [e.g., fibroblast growth factors (FGFs) and epidermal growth factor], chemokines, and a distinct ECM composition that sequesters growth factor signaling and spatially restricts cell movement for appropriate cell–cell interactions. While well-defined niches characterized in different organs vary in size and complexity, most are maintained by a small set of fundamental regulatory signaling loops (51). Although several studies have revealed the importance of these signaling pathways in the adrenal biology as coordinators of stem/progenitor cell specification and differentiation, the formal structural and molecular definition of an adrenocortical progenitor niche remains elusive (52). ECM components are predicted to provide additional structural context to zone-specific signaling pathways discussed in the previous section. Below, we discuss these regulatory mechanisms in further details.

### Components of the Adrenocortical Niche

#### Soluble Factors

##### Wnt Ligands

As previously discussed, the canonical Wnt/β catenin signaling pathway is considered to be a crucial pathway for the maintenance of the subcapsular stem/progenitor pool (44, 46, 53). While the source and the contribution of different Wnt ligands to Wnt signaling are not currently known, previous observations based on adrenal tumors expression profiles, human clinical syndromes, and mouse models suggest that WNT4 is an important Wnt regulatory paracrine factor in the adrenal cortex. In humans, *WNT4* mutations underlie the defects in Serkal syndrome, an autosomal recessive disorder characterized by multiple malformations including adrenal hypoplasia and male-to-female sexual reversion (54). In mice, while Wnt4 expression is observed in the developing adrenal glands as early as E11.5, by E14.5 it is restricted to the outer cortex (46, 55). Additionally, a mouse model of Wnt4 inactivation suggests that this ligand is required for proper zG differentiation and aldosterone production. In these mice, genetic loss of *Wnt4* decreases *Cyp11b2* expression, leading to a diminished production of aldosterone (55). Interestingly, the anterior tips of the developing gonads of these mice exhibit a steroidogenic enzyme expression pattern similar to adrenocortical cells, suggesting an abnormal differentiation process or a defect in adrenal cell specification during the separation of the AGP (55, 56).

*WNT4* is a known transcriptional target of Wnt/β catenin signaling (55). In the adrenal cortex, WNT4 expression is restricted to the zG, precisely where canonical Wnt pathway is known to be active. Additionally, according to our own analysis on a recently published molecular profiling study on adrenocortical carcinomas (57), samples with activating exon 3 *CTNNB1* mutations exhibit increased expression of *WNT4* in comparison to samples without somatic alterations in components of the Wnt pathway (fold change = 17.5, FDR-adjusted *p*-value <0.001). More recently, it has been demonstrated that the expression levels of canonical Wnt target genes are significantly reduced in the adrenals of a mouse model of *Sf1-Cre* mediated *Wnt4* knockout (*Sf1:Cre;Wnt4Fl/Fl)* (58). Interestingly, it has also been shown that the activation of the protein kinase A pathway enables a zG-to-zF lineage conversion by antagonizing canonical Wnt signaling, which is partially mediated by repression of *Wnt4* expression (58). Together, these data support a model whereby WNT4 serves as an autocrine activator of Wnt signaling, amplifying canonical (β catenin-dependent) Wnt activation within the zG, which is essential for proper zonation. However, whether WNT4 downstream signaling also induces a non-canonical (β catenin-independent) effect is currently not known. In other systems, WNT4 is described preferentially as a non-canonical ligand (59, 60). Last, the roles and the sources of other Wnt ligands that are expressed in the adrenal are yet to be determined.

##### Other Wnt Pathway-Related Soluble Factors

Bone Morphogenetic Protein-4 (BMP4) is a ligand of the TGF-β superfamily that plays essential roles in embryonic development, stem cell biology, and tissue regeneration (61). Recently, Rege et al. have demonstrated that this protein, its receptors, and downstream molecules are expressed and have functional roles in the human adrenal cortex and in the H295R adrenocortical carcinoma cell line. Interestingly, mRNA expression of *BMP4* follows a zonal distribution gradient, with the highest levels in the zG, suggesting that *BMP4* is a Wnt target gene. *In vitro*, BMP4 actively inhibits the expression of 17,20-lyase and DHEA secretion, possibly serving to prevent a zR fate (62). Consistent with the observed zonal distribution in the adrenal cortex, *BMP4* has been reported as a Wnt target gene in a colorectal cancer cell line (63). Moreover, according to our own analysis on two independent datasets (57, 64), *BMP4* mRNA is upregulated in ACC samples with nuclear immunostaining for β-catenin or with an activating mutation of *CTNNB1* in comparison to those with membranous immunostaining or wild-type for *CTNNB1* somatic mutations (fold change >3.21–6.76, FDR-adjusted *p*-value <0.001). These observations suggest that *BMP4* is a paracrine factor regulated by the Wnt pathway that may have a role in functional zonation by inhibiting a zR fate.

Coiled-coil domain containing 80 (*CCDC80*) has recently been described as a novel Wnt target gene in the adrenal cortex. *In situ* hybridization has shown that it is expressed in cells within the zG. Functional studies have shown that CCDC80 decreases steroidogenesis *in vitro*, suggesting, as for BMP4, a role in cell differentiation or fate. The molecular pathway by which CCDC80 regulates steroidogenesis is yet to be determined in the adrenal gland (44). In the chick embryo, the *CCDC80* homolog equarin regulates FGF signaling by increasing its local availability and by facilitating the interactions of FGF ligands with FGF receptors and proteoglycans (65). Further studies in *CCDC80* null mice aiming to characterize the overall mechanism of action of *CCDC80* in the adrenal gland are ongoing.

##### Sonic Hedgehog

Sonic hedgehog-expressing cells serve as a *bona fide* progenitor population that contributes to the adrenal cortex homeostasis in adult life, as previously described. Mice with global genetic deletion of *Shh* exhibit severe adrenal hypoplasia during embryonic development due to a reduction in both cortical and capsular cell proliferation (8, 66, 67). However, these animals also have severe malformations in other organs, including pituitary defects that impair ACTH production, making the adrenal phenotype difficult to interpret. Moreover, targeted deletion of *Shh* in Sf1-expressing cells in mice also causes adrenal abnormalities, indicating that Shh is indeed essential for intrinsic (ACTH-independent) adrenal development. While AGP separation, neural crest migration, and encapsulation occur normally, these animals have a hypoplastic adrenal cortex and diminished mitotic activity by E13.5. As age increases, progressive cortical thinning with an apparent defect in steroidogenesis is observed as reflected by a compensatory increase in ACTH levels. Also, an overall thinner capsule is also seen (66). The mechanisms by which Shh deficiency causes this capsular phenotype are not known, but is predicted to result from a defective cortical Shh signaling to the capsular Gli1-expressing cells.

Shh is a secreted ligand that binds to the cell-surface receptor patched homolog 1 (Ptch1). Shh binding relieves Ptch1-mediated inhibition of Smoothened homolog (Smo). Smo stimulates intracellular downstream Shh signaling that, when activated, inhibits the otherwise proteolytic degradation of Gli transcription factors, resulting in Gli-mediated transcriptional activation of Hh target genes (8, 47, 66, 67). Whether a secreted factor produced by Shh-responsive Gli+ capsular cells is required for maintaining the subcapsular progenitor population is currently unknown. Together, these observations indicate that the Shh-expressing subcapsular progenitor cells and the capsular Gli-expressing cells are integral components of the adrenocortical progenitor cell niche.

##### Fibroblast Growth Factors

The FGFs are a family of secreted signaling proteins that bind to a subclass of membrane-bound tyrosine kinase-coupled receptors (FGFRs 1–4) and intracellular non-signaling proteins (iFGFs) that work as cofactors for other membrane-associated proteins. With few exceptions, FGFs are ubiquitous paracrine/autocrine factors with essential roles in embryogenesis, organogenesis, and tissue homeostasis. FGFs regulate fundamental cellular processes, including proliferation (both promoting and inhibiting), survival, differentiation, migration, and metabolism. FGFs routinely bind to heparin sulfate proteoglycans, which limit their diffusion through the ECM and serve as cofactors that regulate FGF specificity and affinity to different types of FGFRs (68, 69).

Early studies have demonstrated that FGFs exhibit a potent mitogenic effect both in the murine-derived Y1 adrenocortical cell line and primary cultures of bovine and human adrenocortical cells. Interestingly, this effect can be antagonized by ACTH, which promotes cell differentiation by inducing cell cycle arrest and steroidogenesis *in vitro* (70–74). *In vivo*, different combinations of FGF ligands and receptors are expressed in the adrenal capsule and cortex, consistent with a proposed role in homeostasis. RT-PCR analysis from laser capture microdissected adrenals from E15.5 mouse embryos have shown that *Fgf1, Fgf2*, and *Fgf9* are the only FGF ligands that are expressed in the embryonic adrenal gland. While *Fgf1* is expressed in the cortex, *Fgf2* and *Fgf9* are expressed preferentially in the capsule. Additionally, *Fgfr1*-IIIc, *Fgfr2*-IIIb, and *Fgfr2*-IIIc are expressed in cells of both the capsule and the cortex. Interestingly, the cortical cells that express *Fgfr2* are the also Shh-positive (48, 75, 76).

The importance of FGF signaling in adrenal maintenance has been revealed through several *in vivo* studies. FGF2 encapsulated in poly-lactic-co-glycolic acid enhances the growth of adrenocortical cells implanted under the kidney capsule of mice by fivefold to eightfold (74). Additionally, engineered deletion of the FGF ligand, FGF2 or the FGF receptor *Fgfr2*, results in various degrees of adrenal hypoplasia after birth. The global *Fgfr2-*IIIb knockout results in embryonic lethality due to severe malformations, including adrenal hypoplasia (7). The same adrenal phenotype was recapitulated by a *Sf1*-driven specific knockout of both *Fgfr2-*IIIb and *Fgfr2-*IIIc (77). Evaluation of the embryonic adrenal gland of *Fgfr2*-IIIb global knockout mice at E15.5 revealed a marked capsular defect, characterized by thickening and disorganization of the mesenchymal capsule, with increased mitotic activity and increased number of Gli1-positive cells. Meanwhile, the underlying cortex was hypoplastic with a decrease in both steroidogenic differentiation and mitotic activity. An unexpected, decrease in capsular delta-like protein 1 (Dlk1) suggests that, together with Shh and FGFs, Dlk1 is a mediator of the proposed homeostatic cross-talk between cortical and capsular cells (75).

##### Delta-Like Protein 1 (DLK1)

Delta-like protein 1 (also known as preadipocyte factor 1*—Pref1*) is a paternally expressed imprinted gene that encodes an epidermal growth factor repeat-containing transmembrane protein. Dlk1 is cleaved by the TNF-alpha-converting enzyme (TACE or ADAM17) to generate a soluble, secreted, and biologically active protein (78, 79). In adipose tissue, DLK1 negatively regulates proliferation and terminal differentiation of adipocyte progenitors (80–83). Mechanistically, DLK1 interacts with fibronectin and facilitates downstream integrin signaling, which includes MEK/ERK activation (80). While the human adrenal cortex expresses high levels of the *DLK1* transcript (the highest levels among all human tissues according to the GTEx portal database), little is known about its downstream signaling and overall function in adrenocortical cells (84). However, *in vivo* studies in rat reveal that *Dlk1* expression is markedly decreased in the regeneration process following enucleation, suggesting a role in adrenal remodeling and zonation. The Dlk1 expression is restricted to a subcapsular population in the rat zU (6, 85, 86). Indeed, the Dlk1+ cells do not express Cyp11b1 and are rarely positive for Cyp11b2. They do, however, express Shh, indicating that they are indeed the progenitor population (48, 87). A low-sodium diet induces a marked downregulation of both Dlk1 and Shh, with a proportional increase in the expression of Cyp11b2. Blocking the RAAS with captopril induces the opposite effect—expansion of Dlk1 and Shh-expressing cells. Additionally, a population of capsular cells responds directly to Dlk1 signaling by increasing the levels of both p-ERK and Gli1, consistent with a role in regulating hedgehog signaling (6). These results predict that in the rat adrenal, Dlk1 is expressed by the cortical progenitor cell population and acts in concert with Shh to activate capsular Gli1, modulating differentiation, remodeling, and zonation (6). However, it should be noted that these conclusions are based on studies in rat, which express Dlk1 in the subcapsular cells whereas, in mice, Dlk1/Pref1 is expressed in the capsule (6, 75).

##### R-Spondin Family Member 3 (RSPO3)

R-spondins are secreted proteins that have been recently described as important positive regulators of the canonical Wnt pathway (88). R-Spondins are paracrine factors that exhibit a very low diffusion gradient since these proteins firmly binds to certain ECM components such as syndecan-4, remaining close to its secretory source (89). In organs, such as the liver and the gastrointestinal tract, RSPO3 gradients are a critical determinant of regions of active canonical Wnt signaling, being a crucial regulator of the stem/progenitor cell compartment, cell differentiation, and functional compartmentalization (90). R-Spondins interact with members of the leucine-rich-repeat-containing G-protein-coupled receptor (Lgr), which have recently been described as surface stem cell markers (91). Clinical evidence suggests an important role of R-Spondins in stem cell biology and morphogenesis since inactivating mutations in R-Spondin genes are associated with syndromes characterized by stem cell failure and developmental abnormalities (89). The interaction of R-Spondins with the Lgr receptors results in the inactivation of the U3-ubiquitin ligases RNF43 and ZNRF3, which are negative regulators of Wnt signaling by promoting internalization of the *Frizzled* receptors. Consequently, in the presence of R-Spondins, the *Frizzled* receptor will remain on the membrane allowing for Wnt signaling activation (91, 92). A recent paper explores the roles of R-Spondins in the regulation of the canonical Wnt signaling, functional zonation, and progenitor cell activity in the adrenal cortex (5). By using *in situ* hybridization, the authors demonstrated the expression of *Rspo1* and *Rspo3* in the adrenal capsule of the mouse from E12.5 onward. While *Rspo1* co-localized with the *Wt1* mesodermal-like expressing cells, *Rspo3* was preferentially co-expressed with *Nr2f2* and *Gli1*-expressing cells. Additionally, the mRNA abundance of *Rspo3* was significantly higher than *Rspo1*. Interestingly, while the genetic loss of *Rspo1* had no observable effects on the adrenals, loss of *Rspo3* was associated with remarkable phenotypes. By using different crosses of a *Rspo3^flox^* allele and several CreERT drivers, the authors demonstrated that (a) loss of *Rspo3* results in cortical atrophy both during development and postnatally; (b) these changes are accompanied by a dramatic decrease in canonical Wnt signaling, which results in loss of the expression of canonical Wnt target genes such as *Axin2* and *Wnt4*; (c) loss of a functional zG, characterized by profound morphological changes and loss of expression of zG markers (while retaining zF differentiation); (d) loss of cortical Shh-expressing cells and capsular Gli1-expressing cells; and (e) a significant decrease in the mitotic activity of the cortex. The authors concluded that capsular-derived Rspo3 is a key regulator of both Wnt and Hedgehog signaling in the adrenal cortex, having determinant roles in the establishment and the maintenance of the stem/progenitor cell populations, and in functional zonation (5).

**Figure 1:**
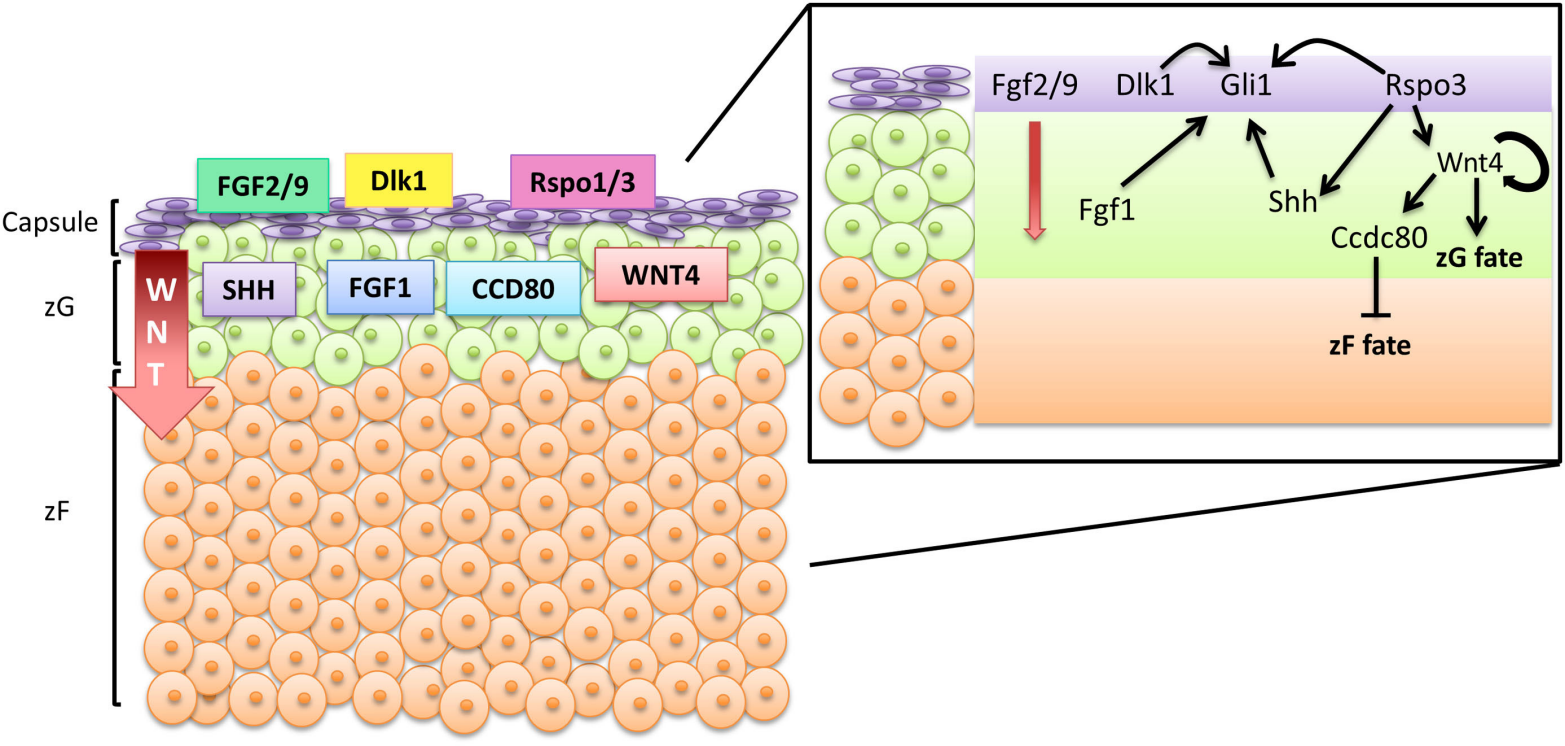
**Schematic figure representing paracrine signaling in the adrenal cortex**. Adrenal zonation is established and maintained by a compartmentalized secretion of paracrine factors. The zG is characterized by activation of canonical Wnt signaling, which is dependent on the presence of RSPO3 secreted by Gli1-expressing capsular cells. Wnt-expressing cells secrete other paracrine factors that regulate the differentiation of cortical cells. Wnt4 amplifies the activation of canonical Wnt signaling within the zG, helping in maintaining the zonation. Ccdc80 and is a canonical Wnt target gene that prevents zF differentiation. By unknown mechanisms, a small proportion of Wnt-active zG cells, which is a long-living progenitor cell population, secretes Shh, which activates Gli1-dependent signaling on the capsular cells. Other paracrine factors, such as Dlk1 and fibroblast growth factors, also have regulatory roles in the maintenance of the cortical progenitor cells, presumably by regulating Gli1 expression in the capsule.

These above findings support a general model whereby several autocrine/paracrine mediators from different sources play regulatory roles in the major signaling pathways that control the progenitor cell compartment and fate determination in the adrenal cortex (Figure 1). Some are known to interact with ECM components, indicating that the ECM composition may play a role in these regulatory loops. However, several gaps in the knowledge about the role of the adrenal ECM in paracrine/autocrine signaling and cell fate determination remain. In the following sections, we summarize the main findings that support a regulatory role for the adrenal ECM.

#### The ECM and Associated Proteins

The ECM is a three-dimensional meshwork of extracellular proteins and polysaccharides that supports cells in each tissue (93). Among its components are included fibrous proteins such as collagens, laminin, fibronectin, and several types of bioactive compounds, such as latent growth factors, enzymes, chemoattractants, and morphogens (94). Therefore, in addition to its role in providing the physical scaffold that shapes the different tissues in an organism, the ECM is a reservoir of several biologically active compounds of different sources that are delivered to the cells in a spatially controlled manner (93, 94). Cells attach to the ECM through a special type of membrane-bound adhesion molecule known as integrins, which are comprised of two interacting subunits. The vertebrate integrin family contains 18 α and 8 β subunits that can assemble into 24 different receptor complexes, each with unique binding properties for different ECM components (93). In the context of the stem cell niche of a variety of organ systems, the interactions between ECM proteins and integrins are fundamental for establishing a balance between self-renewal and differentiation. By forming complexes with different membrane receptors and ECM components, integrins facilitate a strict compartmentalization of cellular responses to different factors on a cell-by-cell basis. Such highly precise control is fundamental to the process of asymmetric division, a fundamental characteristic of self-renewing cells. The complexes that contain integrins, ECM components, and other receptors induce the establishment of cell polarity, restricting fate-determinant molecules to one pole and directing the plane of cell cleavage in such a way that signaling molecules will be asymmetrically distributed between the daughter cells (95). In fact, certain types of integrins have been recognized as crucial for stem cell maintenance in different tissues (96). Supporting this observation, genetic ablation of β1 integrin favors a symmetric pattern of cell division in the mammary stem cell niche, leading to exhaustion of the stem cell compartment (97). Finally, biophysical and biochemical properties of the substrate, such as shape, stiffness, and protein composition, can trigger different transcriptional programs that support either stem cell maintenance or differentiation (98, 99).

Despite the emerging importance of ECM in tissue homeostasis and stem cell biology, few studies have addressed its roles in the adrenal. Early studies in different species have shown that several ECM components and integrins follow a zonal distribution in the adrenal cortex (100, 101). Furthermore, *in vitro* studies on primary adrenal cultures grown on different substrates suggest that different ECM components affect cellular responses to ACTH. For instance, it has been demonstrated in primary cultures from human fetal adrenals that laminin exerts an inhibitory effect on basal and ACTH-induced steroidogenesis, and a positive effect on cell proliferation (102). On the other hand, collagen IV and fibronectin increased cortisol and DHEAS production, respectively (102). While collagen IV is expressed throughout the human fetal adrenal gland, laminin and fibronectin follow a zonal distribution, being preferentially expressed in cells of the definitive and the fetal zones, respectively (101). Therefore, the differences in cellular responses *in vitro* induced by these different ECM components parallel the functional zonation observed in the fetal gland: laminin suppresses differentiation and promotes proliferation (consistent with the cellular phenotype of the newly forming definitive zone), collagen IV promotes *HSD3B2* expression and cortisol production (resembling the fetal adrenal transitional zone), and fibronectin supports *CYP17A1* expression and DHEAS production. Indeed, primary cultures from zG and zF cells from the adult rat adrenal grown on laminin-coated plates exhibited increased proliferation and reduced basal and ACTH-stimulated aldosterone and cortisol production, respectively (103). Taken together, these results support the hypothesis that the ECM composition modulates cellular responses to hormone and growth factor stimulation, providing an additional mechanism by which the niche itself regulates the balance between self-renewal and differentiation.

## Concluding Remarks and Future Perspectives

In the last decade, several important aspects of the physiology of the adrenal cortex have been characterized, including the developmental origins of the different progenitor populations and the molecular pathways that are essential for self-renewal, organ remodeling, and differentiation. Additionally, several paracrine/autocrine bioactive compounds that initiate and maintain these signaling pathways have been identified. However, while the progenitor niche populations of other tissues, such as the skin and the gastrointestinal tract, are clearly defined, the progenitor niche of the adrenal cortex is just beginning to be characterized. Many features of the molecular mechanisms that govern the fate of the adrenocortical progenitor cells are still unknown. As discussed in the previous sections, several studies involving transgenic animal models have illuminated important observations and concepts central to the field. However, these studies are complex and time-consuming. Furthermore, studying such a small yet complex organ like the adrenal gland imposes additional challenges. In the near future, innovative techniques that allow investigators to engage in more complex *in vitro* studies have the potential to move the field forward greatly. Among these, organoid culture and engineered ECM are promising approaches.

Organoid culture has emerged in recent years as a valuable tool to study several aspects of stem cell biology, tissue morphogenesis, and lineage specification (104, 105). Organoids are self-organizing three-dimensional structures that are derived from stem cells and exhibit organotypic anatomic and functional features, including spatially restricted lineage commitment (106, 107). Organoids can be grown *in vitro* from pluripotent stem cells (embryonic stem cells and induced pluripotent stem cells) or organ-specific stem cells (105). Organoid cultures have been established for different mouse and human tissues, including intestines, stomach, lungs, mammary gland, and prostate (108–114). A crucial requirement for establishing an organoid culture is the supplementation of organ-specific and well-defined stem cell niche factors in the culture medium. For example, to culture intestinal organoids, culture medium is supplemented with Wnt3a, EGF, Noggin, and R-spondin 1 (108). Organoids have been proven to be valuable models for studying different aspects developmental biology and stem cell research such as tissue morphogenesis, organogenesis, differentiation, heterotypic interactions between different cell types, and the effects of the ECM on cell differentiation and behavior. However, a current limitation of organoid cultures is the organoid dependence on a suitable three-dimensional matrix. Among the most commonly used scaffolds are collagen and Matrigel. These substrates, however, feature variable compositions and physical properties, which limits studies aiming to characterize the influence of the microenvironment on the organoid properties (105). Furthermore, these substrates pose risks of immunologic reactions and pathogen transfer and are, therefore, unsuitable for clinical applications. Engineered synthetic ECM has emerged as an attractive approach to circumvent these limitations (115).

Engineered ECM, constructed as an artificial substrate, acts to mimic the original three-dimensional microenvironment of a given organ or tissue. Increasingly, sophisticated scaffolds comprised of materials, such as synthetic hydrogels and nanofibers, are frequently employed as artificial ECM substitutes. In addition to biochemical interactions in cell–cell and cell–ECM signaling, mechanical stimuli such as substrate stiffness, and tension forces can also be modeled in engineered ECM (115). In a recent publication Gjorevski et al. studied the effects of stiffness on the dynamics of single cell-derived intestinal organoid formation (116). While intermediate to high stiffness and the presence of fibronectin sustained the expansion of intestinal LGR5-positive stem cells, differentiation and organoid formation required softer matrices and the presence of laminin.

We believe that, in the near future, organoid culture and tissue engineering techniques will increase our power to understand the molecular interactions that regulate the adrenal cortex stem cell niche. Protocols to establish normal adrenocortical and cancer organoid cultures in bioengineered matrices are currently under development in our laboratory.

## Author Contributions

All authors equally contributed to this manuscript.

## Conflict of Interest Statement

The authors declare that the research was conducted in the absence of any commercial or financial relationships that could be construed as a potential conflict of interest.
